# Unilateral and bilateral radioactive stent insertion in patients diagnosed with inoperable hilar cholangiocarcinoma: a comparative analysis

**DOI:** 10.3389/fonc.2024.1412933

**Published:** 2024-10-01

**Authors:** Jin-Long Jin, Wei Li, Zhi-Xian Wu, An-Qiang Feng, Hao Li

**Affiliations:** ^1^ Interventional Department of Peripheral Vascular Disease, Gansu Provincial Hospital of Traditional Chinese Medicine (TCM), Lanzhou, China; ^2^ Department of Radiology, Xuzhou Central Hospital, Xuzhou, China; ^3^ Department of Human Affairs, Gansu Center for Disease Control and Prevention, Lanzhou, China; ^4^ Department of Digestive Disease, Xuzhou Central Hospital, Xuzhou, China; ^5^ Department of Interventional Radiology, Xuzhou Central Hospital, Xuzhou, China

**Keywords:** radioactive, stent, hilar, cholangiocarcinoma, survival

## Abstract

**Background:**

While hilar cholangiocarcinoma (HCCA) patients commonly undergo radioactive stent (RS) insertion treatment, the relative benefits of unilateral versus bilateral RS insertion procedures remain to be established. Accordingly, this study was designed to evaluate the relative safety and efficacy of percutaneous bilateral and unilateral RS insertion for patients with HCCA.

**Methods:**

In total, 126 HCCA patients who underwent unilateral (n=64) or bilateral (n=62) RS insertion from January 2017 - December 2021 were included in this analysis. Treatment efficacy and long-term outcomes were compared between groups. The primary endpoint was stent patency, and the secondary endpoints included technical success rate, clinical success rate, local control rate, overall survival (OS), and complications.

**Results:**

The respective technical success rates in the unilateral and bilateral groups were 90.6% (58/64) and 93.5% (58/62) (P = 0.782). The clinical success rates were 82.8% and 86.2% in unilateral and bilateral groups, respectively (P = 0.608). Both groups exhibited comparable medial post-intervention bilirubin levels (100 vs. 99 μmol/L; P = 0.501), and restenosis occurred in 12 (20.7%) and 15 (25.9%) patients over the follow-up interval (P = 0.510). The stent reintervention rate was significantly higher in the unilateral group than bilateral group (66.7% vs. 0.0%, P < 0.001). The median stent patency in the unilateral and bilateral groups was 189 and 210 days, respectively (P = 0.796), while the median OS interval was 222 and 229 days, respectively (P = 0.969). Comparable cholangitis (17.2% vs. 22.4%, P = 0.485) and cholecystitis (3.4% vs. 3.4%, P = 1.000) rates were also detected in these two groups.

**Conclusions:**

In summary, HCCA patients exhibit comparable efficacy when undergoing unilateral and bilateral radioactive stenting, suggesting that unilateral RS can be routinely performed owing to the simpler nature of this procedure.

## Introduction

Hilar cholangiocarcinoma (HCCA) tumors are malignancies of the hepato-biliary system that can only be surgically resected in under 30% of cases (1). HCCA patients often experience obstructive jaundice and a consequent reduction in their quality of life (2, 3). In these inoperable HCCA patients, the insertion of a metal stent is often used as a first-line approach to alleviating jaundice symptoms (4–6). In 2012, Zhu et al. (7) combined a metal biliary stent and ^125^I seeds to generate a radioactive stent (RS) capable of prolonging stent patency and patient overall survival (OS) owing to the brachytherapeutic effects exerted by persistent ^125^I seed exposure.

While stenting is well-established as a therapeutic approach, controversy persists as to whether unilateral or bilateral stent insertion should be conducted in HCCA patients. Some studies have determined that endoscopic bilateral stent insertion can result in superior liver drainage and the prolongation of stent patency, with lower perioperative mortality rates (8–10). Others, however, have found that the technical success rates, clinical success rates, OS, stent patency, and complication rates associated with using a percutaneous trans-hepatic approach for unilateral or bilateral stent insertion were comparable (11, 12). All of these prior studies also employed traditional metal stents irrespective of whether a percutaneous or endoscopic insertion approach was used (8–13), and a detailed understanding of the relative benefits of unilateral or bilateral RS insertion is currently lacking.

This study was designed to address this knowledge gap by comparing the safety and efficacy of percutaneous unilateral and bilateral RS insertion in individuals diagnosed with HCCA.

## Methods

### Study subjects

This retrospective study was conducted by 2 centers (Gansu Provincial Hospital of TCM and Xuzhou Central Hospital) from January 2017 to December 2021. The local Institutional Review Board of Gansu Provincial Hospital of TCM (No. 2023-131-01) and Xuzhou Central Hospital (No. XZXY-LK-20220519-039) approved the present retrospective analysis, for which the requirement for informed consent was waived. In total, this study included 126 HCCA patients who underwent either unilateral (n=64) or bilateral (n=62) RS insertion (**Table 1**).

**Table 1:** Baseline data of the included patients.

	Unilateral group (n = 64)	Bilateral group (n = 62)	P value
Age (y)	65.0 ± 8.7	63.7 ± 8.2	0.391*
Gender			0.852
Male	33	33	
Female	31	29	
Tumor stage			0.941
III	44	43	
IV	20	19	
Bismuth type			0.280
II	24	32	
III	27	20	
IV	13	10	
ECOG PS	1.4 ± 0.8	1.3 ± 0.9	0.578*
TBIL before treatment (μmol/L)	190 (Q1:123; Q3: 349)	178 (Q1:123; Q3: 267)	0.240^#^
TBIL after treatment (μmol/L)	100 (Q1:39; Q3: 121)	99 (Q1:58; Q3: 116)	0.501^#^
CA19-9 (U/ml)	400 (Q1:153; Q3: 827)	384 (Q1:256; Q3: 1459)	0.043^#^
Post-operative chemotherapy	23	23	0.893

ECOG PS, Eastern Cooperative Oncology Group performance status; TBIL, total bilirubin.

*Independent t-test.

^#^Mann-Whitney U test.

Patients eligible for inclusion were individuals with (i) a histology- or cytopathology-confirmed diagnosis of HCCA, (ii) inoperable disease due to tumor invasion or metastasis, (iii) obstructive jaundice; and (d) an ECOG performance status (PS) ≤ 2. Patients were excluded if they (i) had undergone prior hepatectomy, (ii) suffered from uncontrollable intractable ascites, (iii) were Bismuth type I patients, or (iv) had a life expectancy of less than 3 months.

### Diagnosis

Intraductal biopsy was used to confirm the pathological diagnosis of HCCA in all patients. Abdominal MRI and CT scans were used to assess the patients’ Bismuth type and the degree of biliary obstruction.

### RS production

To prepare an RS, a metal stent (Micro-Tech, Nanjing, China) was combined with an ^125^I seed strand composed of a 4F medical catheter (PBN MEDICALS Denmark A/S, Stenlose, Denmark) and multiple ^125^I seeds (Chinese Atomic Energy Science Institution, Beijing, China). These seeds were placed in a line within the catheter, followed by sealing the ends of the catheter. Each of these seeds (0.8 mm × 4.5 mm) emitted low-dose γ-rays (35.5 keV) and soft X-rays (28.6 keV), and each had 0.80 mCi, a 59.6 day half-life, and a 17 mm radius of effective antitumor activity. Each strand included a number of seeds determined based on the length of the stent as follows: N = stent length (mm)/4.5 + 4 (14).

### RS insertion

**Figure 1:**
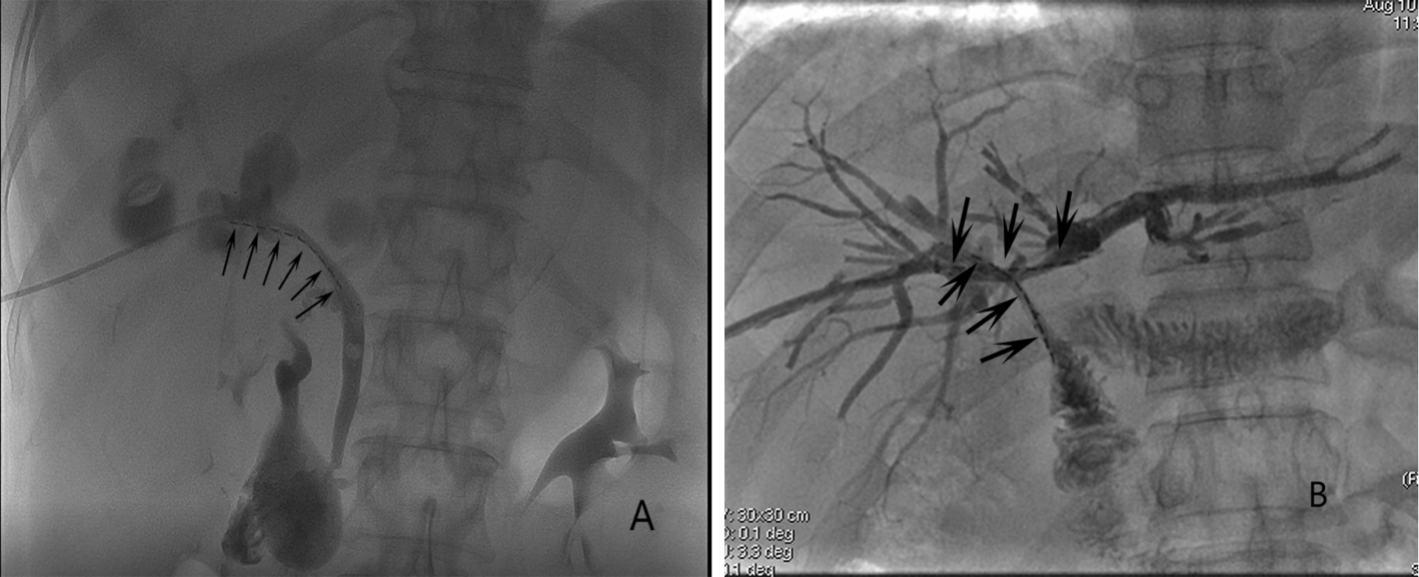
The images of **(A)** unilateral RS insertion (arrows) and **(B)** bilateral RS insertion (arrows).

A percutaneous trans-hepatobiliary tract approach was used for unilateral RS insertion under fluoroscopic guidance. After successfully puncturing the intra-hepatic biliary tract and the placement of a catheter sheath, a 4F catheter (Cordis, FL, USA) was introduced into the biliary tract across the site of the obstruction using a 0.035-inch guide-wire (Terumo, Tokyo, Japan). After the catheter was in the duodenum, this guide-wire was exchanged for a 0.035-inch stiff guide-wire (Cook, IN, USA). Then, another guide-wire was used to introduce a 6F catheter sheath (Terumo) across the obstructed site, and the stiff guide-wire was used to deploy the stent in the center of the obstructed site. ^125^I seeds were placed within this 6F sheath, with the sheat gradually removed so that the seed strand remains positioned between the stent and the biliary wall (**Figure 1A**).

A side-by-side approach was employed for bilateral RS insertion (**Figure 1B**), where the stents were placed in parallel (4). A Y- or T-configuration was selected for stent insertion based on the extent of disease in each HCCA patient.

An 8.5F temporary biliary drainage catheter (Cook) was placed instantly after stent insertion for internal drainage for 5 days, during which all patients underwent hemostatic and anti-inflammatory drug treatment.

### Definitions

Technical success was defined as successful biliary obstruction recanalization without any ^125^I seed strand migration. Clinical success was defined as defined as a pre-intervention reduction by at least 50% in the level of total bilirubin (TBIL) within 2 weeks after stenting (13). The local tumor response was assessed by the response evaluation criteria in solid tumors (RECIST) criteria (**Supplementary Table 1**). Local control was calculated based on the number of patients with complete response, partial response and stable disease (1). Stent restenosis was defined as a recurrence of jaundice and/or cholangitis due to any reason (12). The reintervention was defined as any type of endoscopic or percutaneous procedure necessary to improve biliary drainage for jaundice or cholangitis after stent insertion (10). Stent patency was measured from stent insertion to initial jaundice recurrence. OS was measured from stent insertion to all-cause mortality. In cases where patients died without recurrent jaundice, OS and stent patency were the same. The treatment-related adverse events were classified according to the American Society for Gastrointestinal Endoscopy (ASGE) lexicon (**Supplementary Table 2**) (15).

The primary endpoint was stent patency, and the secondary endpoints included technical success rate, clinical success rate, local control rate, OS, and complications.

### Follow-up

All patients underwent follow-up at 1 week, 1, 3, and 6 months after stenting, and every 6 months after that. Follow-up analyses included physical examinations, liver function tests, and abdominal CT imaging.

### Statistical assessment

Data were analyzed with SPSS 16.0 (SPSS, Inc., IL, USA). Normally distributed data are reported as means with mean ± standard deviation, while they were otherwise presented as medians (Q1; Q3). These results were compared with paired samples t-tests and Wilcoxon tests as appropriate. Categorical variables were compared using χ^2^ tests or Fisher’s exact test. Stent patency and OS rates were compared using Kaplan-Meier curves and the log-rank test. Parameters independently associated with stent patency and OS were identified with a multivariate Cox regression analysis, while parameters significantly related to cholangitis were identified through a multivariate logistic regression approach. P < 0.05 served as the threshold for significance.

## Results

### Technical and clinical success

The respective technical success rates for HCCA patients who underwent unilateral and bilateral RS insertion were 90.6% (58/64) and 93.5% (58/62) (P = 0.782). All stents were 8 mm in diameter and 60-80 mm long. In the 10 cases of technical failure, the guide-wire could not pass through the obstruction site. External drainage catheters were placed in these 10 patients, and stent insertion was again attempted after 1 week. However, the guide-wire remained unable to pass the site of the obstruction in all 10 cases. The clinical success rates were 82.8% and 86.2% in unilateral and bilateral groups, respectively (P = 0.608). Among patients in the unilateral group, the median TBIL declined from 190 μmol/L (Q1: 124, Q3: 348) before stenting to 100 μmol/L (Q1: 39, Q3: 121) after stenting (P < 0.01). Similarly, the median TBIL levels in the bilateral group declined from 182 μmol/L (Q1: 125, Q3: 268) to 99 μmol/L (Q1: 58, Q3: 116) after stenting (P < 0.01). No significant differences in post-intervention TBIL levels were noted between groups (P = 0.501). Chemotherapy was administered following stent insertion for 23 patients in each group.

### Treatment response

In the unilateral group, no patient achieved complete response, 12 (20.7%) patients achieved partial response, 32 (55.2%) patients achieved stable disease, and 14 (24.1%) patients experienced tumor progression. The local control rate was 75.9% (44/58). In the bilateral group, no patient achieved complete response, 11 (20.0%) patients achieved partial response, 37 (63.8%) patients achieved stable disease, and 10 (17.2%) patients experienced tumor progression. The local control rate was 82.8% (48/58). There was no significant difference between 2 groups in the aspect of local control rate (P = 0.359, **Table 2**).

**Table 2:** Treatment effectiveness.

	Unilateral group (n = 64)	Bilateral group (n = 62)	P value
Technical success rate (%)	90.6% (58/64)	93.5% (58/62)	0.782
Clinical success rate (%)	82.8% (48/58)	86.2% (50/58)	0.608
Local control rate (%)	75.9% (44/58)	82.8% (48/58)	0.359
Stent re-stenosis rate (%)	20.7% (12/58)	25.9% (15/58)	0.510
Revision of re-stenosis			< 0.001
Second stent	8	0	
Biliary drainage catheter	4	15	
Stent patency (d)	189	210	0.796
Overall survival (d)	222	229	0.969
Adverse events			
Cholangitis	17.2% (10/58)	22.4% (13/58)	0.485
Cholecystitis	3.4% (2/58)	3.4% (2/58)	1.000

### Stent patency

Of these patients, 12 (20.7%) and 15 (25.9%) experienced stent restenosis throughout follow-up (P = 0.510, **Table 2**), with restenosis resulting from tumor ingrowth in all cases. Of the patients in the unilateral group, 8 underwent the insertion of a second metal stent from the contralateral intra-hepatic biliary tract, and 4 patients underwent biliary drainage catheter insertion. In bilateral group, all 15 patients underwent biliary drainage catheter insertion. No additional reintervention procedure was performed in the patients in both study groups. The stent reintervention rate was significantly higher in the unilateral group than bilateral group (66.7% vs. 0.0%, P < 0.001).

**Figure 2:**
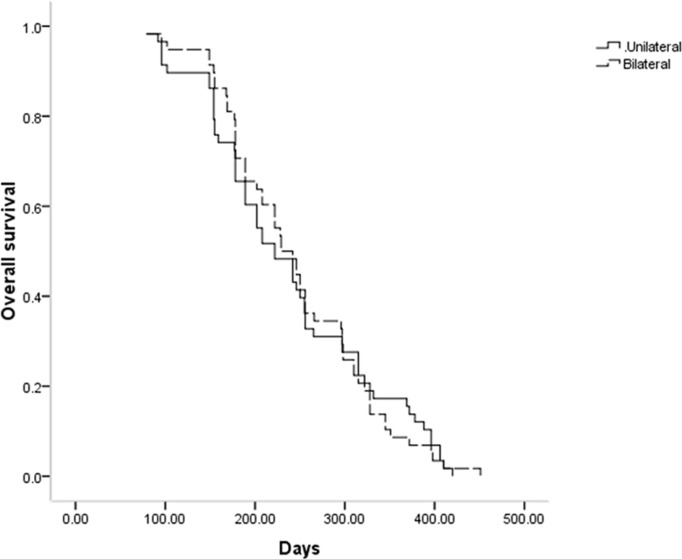
Comparison of stent patency time between the 2 groups.

The median stent patency in the unilateral and bilateral groups was 189 and 210 days, respectively (P = 0.796, **Figure 2**). While univariate analyses revealed that both lower ECOG PS (P = 0.062) and Bismuth type III obstruction (P = 0.067) were related to restenosis, no risk factors associated with stent restenosis were detected through multivariate Cox regression analyses (**Table 3**).

**Table 3:** Predictors of stent patency.

Variables	Univariate analysis			Multivariate analysis		
	Hazard ratio	95% CI	P value	Hazard ratio	95% CI	P value
Age	0.996	0.950-1.044	0.856			
Gender						
Male	1					
Female	1.489	0.689-3.216	0.311			
Tumor stage						
III	1					
IV	0.647	0.244-1.718	0.647			
ECOG PS	0.673	0.445-1.020	0.062	0.668	0.441-1.012	0.057
Bismuth type						
II	1			1		
III	0.362	0.122-1.075	0.067	0.354	0.119-1.052	0.062
IV	0.877	0.325-2.363	0.795	0.827	0.306-2.237	0.709
TBIL before treatment	1.000	0.997-1.004	0.841			
TBIL after treatment	0.993	0.980-1.006	0.302			
Ca19-9	1.000	1.000-1.000	0.511			
Stent insertion						
Unilateral	1					
Bilateral	1.090	0.510-2.331	0.823			
Post-operative chemotherapy						
No	1					
Yes	1.436	0.669-3.081	0.353			

ECOG PS, Eastern Cooperative Oncology Group performance status; TBIL, total bilirubin.

### OS

**Figure 3:**
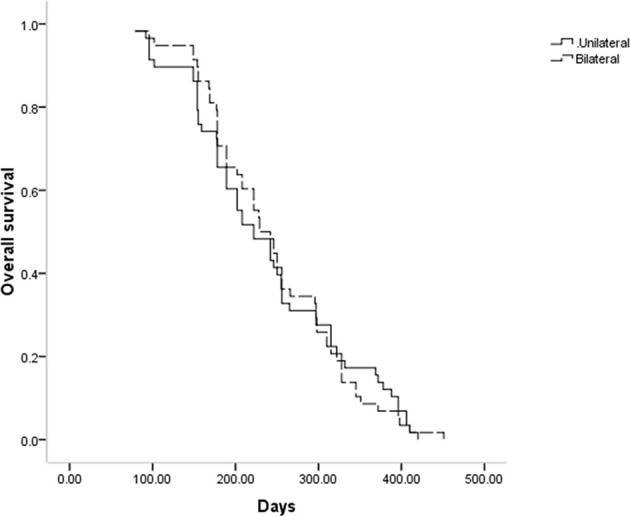
Comparison of OS time between the 2 groups.

Throughout follow-up, all patients included in this study died as a result of tumor progression, with a respective median OS of 222 and 229 days in the unilateral and bilateral groups (P = 0.969, **Figure 3**). Univariate Cox regression analyses identified tumor stage IV (P = 0.070), Bismuth type III obstruction (P = 0.006), and postoperative chemotherapy (P < 0.001) as being associated with shorter patient OS, and multivariate analyses confirmed that tumor stage IV (P = 0.017), Bismuth type III obstruction (P = 0.007), and postoperative chemotherapy (P = 0.006) were all independently associated with the risk of shorter OS (**Table 4**).

**Table 4:** Predictors of overall survival.

Variables	Univariate analysis			Multivariate analysis		
	Hazard ratio	95% CI	P value	Hazard ratio	95% CI	P value
Age	0.993	0.969-1.017	0.549			
Gender						
Male	1					
Female	1.265	0.864-1.853	0.227			
Tumor stage						
III	1			1		
IV	1.471	0.970-2.231	0.070	1.731	1.103-2.716	0.017
ECOG PS	0.916	0.743-1.128	0.408			
Bismuth type						
II	1			1		
III	1.769	1.173-2.667	0.006	1.886	1.186-2.934	0.007
IV	1.013	0.591-1.736	0.963	1.113	0.644-1.925	0.700
TBIL before treatment	0.999	0.998-1.001	0.566			
TBIL after treatment	1.002	0.999-1.006	0.239			
Ca19-9	1.000	1.000-1.000	0.557			
Stent insertion						
Unilateral	1					
Bilateral	0.993	0.688-1.434	0.970			
Post-operative chemotherapy						
No	1			1		
Yes	0.497	0.336-0.736	< 0.001	0.571	0.381-0.854	0.006

ECOG PS, Eastern Cooperative Oncology Group performance status; TBIL, total bilirubin.

### Adverse events

Similar rates of cholangitis (17.2% vs. 22.4%, P = 0.485) and cholecystitis (3.4% vs. 3.4%, P = 1.000) were noted in the unilateral and bilateral groups (**Table 2**). All these adverse events were moderate events according to the ASGE lexicon. Logistic regression analyses failed to identify any cholangitis-related risk factors (**Table 5**).

**Table 5:** Predictors of cholangitis.

Variables	Univariate analysis			Multivariate analysis		
	Hazard ratio	95% CI	P value	Hazard ratio	95% CI	P value
Age	1.048	0.990-1.109	0.104			
Gender						
Male	1					
Female	0.753	0.300-1.888	0.545			
Tumor stage						
III	1					
IV	2.212	0.859-5.697	0.100			
ECOG PS	0.664	0.396-1.114	0.121			
Bismuth type						
II	1					
III	1.091	0.415-2.868	0.860			
IV	0.477	0.095-2.395	0.369			
TBIL	1.000	0.996-1.004	0.994			
Ca19-9	1.000	0.999-1.000	0.236			
Stent insertion						
Unilateral	1					
Bilateral	1.387	0.553-3.477	0.486			
Post-operative chemotherapy						
No	1					
Yes	0.772	0.298-2.001	0.594			

ECOG PS, Eastern Cooperative Oncology Group performance status; TBIL, total bilirubin.

### Subgroup analyses

There were 24 and 32 Bismuth type II patients who underwent unilateral and bilateral RS insertion in this study. The technical (95.8% vs. 93.8%, P = 1.000), clinical success (78.3% vs. 83.3%, P = 0.910), local control (78.3% vs. 80.0%, P = 1.000), and restenosis (34.8% vs. 33.3%, P = 0.912) rates were all comparable between 2 groups. The median stent patency (222 d vs. 225 d, P = 0.344) and OS (297 d vs. 229 d, P = 0.488) were both comparable between 2 groups. The adverse event rates were comparable between 2 groups (21.7% vs. 20.0%, P = 1.000).

There were 40 and 30 Bismuth type III-IV patients who underwent unilateral and bilateral RS insertion in this study. The technical (87.5% vs. 93.3%, P = 0.690), clinical success 85.7% vs. 89.2%, P = 0.723), local control (74.3% vs. 85.7%, P = 0.265), and restenosis (11.4% vs. 17.9%, P = 0.717) rates were all comparable between 2 groups. The median stent patency (189 d vs. 208 d, P = 0.299) and OS (202 d vs. 222 d, P = 0.588) were both comparable between 2 groups. The adverse event rates were comparable between 2 groups (22.9% vs. 25.0%, P = 0.843).

## Discussion

This study was designed to evaluate the safety and efficacy of percutaneous bilateral and unilateral RS insertion approaches in patients diagnosed with HCCA. Both of these procedures exhibited comparably high technical success rates in this analysis (90.6% vs. 93.5%, P = 0.782), confirming the feasibility of both procedures for treating HCCA.

Unlike the previous studies which focused on the bilateral and unilateral conventional metal stent insertion for HCCA (8–12), this present study used the RS instead of the conventional stent. The biliary RS was developed in 2012 with the aim of combining the advantages of rapid relief of the jaundice with stent insertion and brachytherapy with ^125^I seeds (7). Therefore, compared to the conventional stent, RS can decrease the restenosis rate and prolong the stent patency (14).

In many cases, endoscopic guided biliary stent insertion is preferred because it avoids hepatic puncture and allows for physiological biliary tract drainage into the intestine (16, 17). While the technical and clinical success rates associated with stent insertion under endoscopic guidance and via the percutaneous trans-hepatic approach are comparable in cases of distal biliary obstruction, the complication rate associated with the endoscopic approach is notably lower (16, 17). However, endoscopic biliary stenting is inferior to percutaneous trans-hepatic biliary stenting in cases of malignant hilar obstruction, particularly for Bismuth type III/IV obstructions (18, 19). A percutaneous trans-hepatic approach is also important in cases of RS insertion to allow for the insertion of ^125^I seeds through a catheter sheath in the intra-hepatic biliary tract (14).

Theoretically, bilateral stent insertion should yield two different passages for biliary drainage that should be able to drain more bile and prolong stent patency (20, 21). Here, TBIL levels were found to decrease significantly following RS insertion irrespective of the approach employed, with comparable postoperative TBIL values and restenosis rates in the unilateral and bilateral groups, suggesting that these two procedures can yield similar levels of clinical efficacy. These findings may be attributed to the following factors: (a) unilateral stenting can effectively alleviate jaundice in HCCA patients via the drainage of > 25% of bile from the liver, thus providing clinical success (20, 21); (b) using RS instead of traditional metal stents may have inhibited restenosis owing to the brachytherapeutic effects of these stents and the associated inhibition of tumor growth, which is the primary driver of restenosis (22). Moreover, unilateral RS insertion may be superior to bilateral RS insertion in the aspect of the higher successful rate of reintervention for stent restenosis (23).

Both patient groups in this study exhibited comparable OS outcomes, likely owing to the similar stent patency observed in these groups. While RS insertion can suppress tumor growth, this is a palliative treatment strategy rather than a curative one. The local nature of RS brachytherapy also limits its efficacy in treating metastases to lymph nodes or distant sites (24). Here, postoperative chemotherapy was identified as a significant predictor of prolonged patient OS. Chemotherapy can enhance ^125^I seed brachytherapy-related treatment efficacy (25), facilitating the more effective management of lymph nodes and distant metastatic lesions.

Cholangitis is the most important major complication associated with biliary stenting. Deviere et al. (26) reported lower rates of cholangitis following bilateral stent insertion, potentially owing to the more effective drainage of bile than that achieved via the unilateral stent insertion approach. In contrast, Chen et al. (21) posited that the side-by-side insertion of a pair of stents into the common biliary tract can contribute to greater biliary wall compression that may, in turn, exacerbate the cholangitis risk in these patients. No differences in cholangitis were noted between the two RS insertion groups in the present study, nor were any predictors of cholangitis risk successfully identified through logistic analyses. This may be because a temporary biliary drainage catheter was employed following stent insertion, enabling better clearance of the biliary tract in these patients.

There are several limitations to this study. For one, this was a retrospective analysis. Differences in Ca19-9 levels among groups may have also contributed a substantial amount of bias, although no association was noted between Ca19-9 levels and either OS or stent patency. Moreover, the sample size for this study was relatively small, potentially explaining why no risk factors associated with cholangitis or stent patency were identified. Lastly, postoperative chemotherapy was performed in a manner dependent on the condition and consent of each patient, potentially introducing selection bias into these results.

## Conclusion

In summary, these data demonstrate that percutaneous unilateral and bilateral RS insertion procedures can provide HCCA patients with comparable clinical efficacy and long-term outcomes. As a simpler approach, the unilateral RS insertion procedure can thus be routinely recommended in the clinic.

## Data Availability

The raw data supporting the conclusions of this article will be made available by the authors, without undue reservation.

## References

[1] Zhang C Song M Sun Z Fang Y Liu Y Xu K. Biliary drainage combined with simultaneous 125I seed strand brachytherapy for the treatment of hilar cholangiocarcinoma. BMC Cancer. (2023) 23(1):418. doi: 10.1186/s12885-023-10868-5 37161422 PMC10169480

[2] Moll CF de Moura DTH Ribeiro IB Proença IM do Monte Junior ES Sánchez-Luna SA. Endoscopic Biliary Darinage (EBD) versus Percutaneous Transhepatic Biliary Drainage (PTBD) for biliary drainage in patients with Perihilar Cholangiocarcinoma (PCCA): A systematic review and meta-analysis. Clinics (Sao Paulo). (2023) 78:100163. doi: 10.1016/j.clinsp.2022.100163 36681067 PMC10757298

[3] Chen GF Yu WD Wang JR Qi FZ Qiu YD. The methods of preoperative biliary drainage for resectable hilar cholangiocarcinoma patients: A protocol for systematic review and meta analysis. Med (Baltimore). (2020) 99(21):e20237. doi: 10.1097/MD.0000000000020237 PMC7249990 32481299

[4] Zhou WZ Liu S Yang ZQ Xian YT Xu HD Wu JZ. Percutaneous stent placement for Malignant hilar biliary obstruction: side-by-side versus stent-in-stent technique. BMC Gastroenterol. (2020) 20(1):174. doi: 10.1186/s12876-020-01316-w 32503426 PMC7275544

[5] Jiao D Huang K Zhu M Wu G Ren J Wang Y. Placement of a newly designed Y-configured bilateral self-expanding metallic stent for hilar biliary obstruction: A pilot study. Dig Dis Sci. (2017) 62(1):253–63. doi: 10.1007/s10620-016-4284-1 27586033

[6] Lee TH Moon JH Park SH. Biliary stenting for hilar Malignant biliary obstruction. Dig Endosc. (2020) 32:275–86. doi: 10.1111/den.13549 31578770

[7] Zhu HD Guo JH Zhu GY He SC Fang W Deng G. A novel biliary stent loaded with (125)I seeds in patients with Malignant biliary obstruction: preliminary results versus a conventional biliary stent. J Hepatol. (2012) 56(5):1104–11. doi: 10.1016/j.jhep.2011.12.018 22266605

[8] Yang F Wang XM Xia FF Han XQ. Endoscopic metal stenting for Malignant hilar biliary obstruction: an update meta-analysis of unilateral versus bilateral stenting. Wideochir Inne Tech Maloinwazyjne. (2021) 16(3):472–81. doi: 10.5114/wiitm.2021.104196 PMC8512509 34691298

[9] Ashat M Arora S Klair JS Childs CA Murali AR Johlin FC. Bilateral vs unilateral placement of metal stents for inoperable high-grade hilar biliary strictures: A systemic review and meta-analysis. World J Gastroenterol. (2019) 25(34):5210–9. doi: 10.3748/wjg.v25.i34.5210 PMC6747295 31558868

[10] Fu YF Xu YS Shi YB Zong RL Cao C. Percutaneous metal stenting for Malignant hilar biliary obstruction: a systematic review and meta-analysis of unilateral versus bilateral stenting. Abdom Radiol (NY). (2021) 46(2):749–56. doi: 10.1007/s00261-020-02643-y 32671439

[11] Yin X Li DM Yang F Liu TG Xia FF Fu YF. Self-expanded metallic stent insertion for hilar cholangiocarcinoma: comparison of unilateral and bilateral stenting. J Laparoendosc Adv Surg Tech A. (2019) 29(12):1501–6. doi: 10.1089/lap.2019.0509 31553270

[12] Chang G Xia FF Li HF Niu S Xu YS. Unilateral versus bilateral stent insertion for Malignant hilar biliary obstruction. Abdom Radiol (NY). (2017) 42(11):2745–51. doi: 10.1007/s00261-017-1174-8 28477177

[13] Samanta J Sundaram S Dhar J Mane K Gupta P Gupta V. EUS-guided biliary drainage in patients with moderate-severe cholangitis is safe and effective: a multi-center experience. Surg Endosc. (2023) 37:298–308. doi: 10.1007/s00464-022-09495-1 35941304

[14] Zhou C Li H Huang Q Wang J Gao K. Biliary self-expandable metallic stent combined with Iodine-125 seeds strand in the treatment of hilar Malignant biliary obstruction. J Int Med Res. (2020) 48(4):300060519887843. doi: 10.1177/0300060519887843 31884851 PMC7783887

[15] Cotton PB Eisen GM Aabakken L Baron TH Hutter MM Jacobson BC. A lexicon for endoscopic adverse events: report of an ASGE workshop. Gastrointest Endosc. (2010) 71:446–54. doi: 10.1016/j.gie.2009.10.027 20189503

[16] Giri S Seth V Afzalpurkar S Angadi S Jearth V Sundaram S. Endoscopic ultrasound-guided versus percutaneous transhepatic biliary drainage after failed ERCP: A systematic review and meta-analysis. Surg Laparosc Endosc Percutan Tech. (2023) 33(4):411–9. doi: 10.1097/SLE.0000000000001192 37314182

[17] Hassan Z Gadour E. Percutaneous transhepatic cholangiography vs endoscopic ultrasound-guided biliary drainage: A systematic review. World J Gastroenterol. (2022) 28:3514–23. doi: 10.3748/wjg.v28.i27.3514 PMC9346459 36158274

[18] Paik WH Park YS Hwang JH Lee SH Yoon CJ Kang SG. Palliative treatment with self-expandable metallic stents in patients with advanced type III or IV hilar cholangiocarcinoma: a percutaneous versus endoscopic approach. Gastrointest Endosc. (2009) 69(1):55–62. doi: 10.1016/j.gie.2008.04.005 18657806

[19] Saluja SS Gulati M Garg PK Pal H Pal S Sahni P. Endoscopic or percutaneous biliary drainage for gallbladder cancer: a randomized trial and quality of life assessment. Clin Gastroenterol Hepatol. (2008) 6(8):944–950.e3. doi: 10.1016/j.cgh.2008.03.028 18585976

[20] Li M Wu W Yin Z Han G. Unilateral versus bilateral biliary drainage for Malignant hilar obstruction: a systematic review and meta-analysis. Zhonghua Gan Zang Bing Za Zhi. (2015) 23(2):118–23. doi: 10.3760/cma.j.issn.1007-3418.2015.02.009 PMC12769867 25880978

[21] Chen ZK Zhang W Xu YS Li Y. Unilateral versus side-by-side metal stenting for Malignant hilar biliary obstruction: A meta-analysis. J Laparoendosc Adv Surg Tech A. (2021) 31(2):203–9. doi: 10.1089/lap.2020.0400 32644848

[22] Lin LW Ke K Chen R Yang WZ Huang N Wu ZZ. Safety and efficacy of biliary stenting combined with iodine-125 seed strand followed by hepatic artery infusion chemotherapy plus lenvatinib with PD-1 inhibitor for the treatment of extrahepatic cholangiocarcinoma with Malignant obstructive jaundice. Front Immunol. (2024) 14:1286771. doi: 10.3389/fimmu.2023.1286771 38288113 PMC10822914

[23] Yasuda I Mukai T Moriwaki H. Unilateral versus bilateral endoscopic biliary stenting for Malignant hilar biliary strictures. Dig Endosc. (2013) 25 Suppl 2:81–5. doi: 10.1111/den.12060 23617655

[24] Bi Y Li J Yi M Yu Z Han X Ren J. Self-expanding segmental radioactive metal stents for palliation of Malignant esophageal strictures. Acta Radiol. (2020) 61(7):921–6. doi: 10.1177/0284185119886315 31744304

[25] Hong J Shi YB Fu YF Yang LL. Iodine-125 seeds insertion with trans-arterial chemical infusion for advanced lung cancer: a meta-analysis. J Contemp Brachytherapy. (2022) 14(4):403–10. doi: 10.5114/jcb.2022.118117 PMC9528826 36199950

[26] Deviere J Baize M de Toeuf J Cremer M. Long-term follow-up of patients with hilar Malignant stricture treated by endoscopic internal biliary drainage. Gastrointest Endosc. (1988) 34(2):95–101. doi: 10.1016/S0016-5107(88)71271-7 2835282

