# Supplementary material for: Unilateral and bilateral radioactive stent insertion in patients diagnosed with inoperable hilar cholangiocarcinoma: a comparative analysis
Source: Front Oncol. 2024 Oct 1;14:1412933. doi: 10.3389/fonc.2024.1412933 (PMC11473397; doi:10.3389/fonc.2024.1412933)
Supplement: Supplementary file 1 [file Table1.doc]

Table 1 Response Evaluation Criteria in Solid Tumors.

|  | Definitions |
| --- | --- |
| Complete response | Complete disappearance of local tumor |
| Partial response | Maximum transverse tumor diameter decreased by 30% |
| Stable disease | Any cases that do not qualify for either partial response or progressive disease |
| Progression disease | Maximum transverse tumor diameter enlarged by 20% |

Table 2 Severity grading system.

| Consequence | Severity grade | | | |
| --- | --- | --- | --- | --- |
|  | Mild | Moderate | Severe | Fatal |
| Procedure aborted (or not started) because of an adverse event | * |  |  |  |
| Postprocedure medical consultation | * |  |  |  |
| Unplanned anesthesia/ventilation support, ie endotracheal intubation during conscious sedation^#^ |  | * |  |  |
| Unplanned hospital admission or prolongation of hospital stay for ≤ 3 nights | * |  |  |  |
| Unplanned admission or prolongation for 4-10 nights |  | * |  |  |
| Unplanned admission or prolongation for > 10 nights |  |  | * |  |
| ICU admission for 1 night |  | * |  |  |
| ICU admission > 1 night |  |  | * |  |
| Transfusion |  | * |  |  |
| Repeat endoscopy for an adverse event |  | * |  |  |
| Interventional radiology for an adverse event |  | * |  |  |
| Interventional treatment for integument injuries |  | * |  |  |
| Surgery for an adverse event |  |  | * |  |
| Permanent disability (specify) |  |  | * |  |
| Death |  |  |  | * |

#: Temporary ventilation support by bagging or nasal airway during conscious sedation, and endotracheal intubation during a modified anesthesia care procedure are not adverse events.

ICU: intensive care unit.
